# Citrulline Effect Is a Characteristic Feature of Deiminated Peptides in Tandem Mass Spectrometry

**DOI:** 10.1007/s13361-019-02271-x

**Published:** 2019-07-12

**Authors:** Arnold Steckel, Gitta Schlosser

**Affiliations:** 1 Hevesy György PhD School of Chemistry, ELTE Eötvös Loránd University, Budapest, Hungary; 2 MTA-ELTE Research Group of Peptide Chemistry, Department of Organic Chemistry, ELTE Eötvös Loránd University, Budapest, Hungary; 3 Department of Analytical Chemistry, ELTE Eötvös Loránd University, Pázmány Péter sétány 1/A, Budapest, 1117 Hungary

**Keywords:** Citrulline effect, Citrullination, MS/MS, Post-translational modification, Proline effect

## Abstract

Tandem mass spectrometry of peptides is of utmost importance in proteomics. Collision-induced dissociation usually generates *y* type fragment ion series from tryptic peptides, carrying information on their primary structure. Amino acid side chains or differences in their basicity could alter fragmentation processes considerably. The well-known proline effect is a cleavage preference at the *N*-terminus of proline residues in peptides, usually yielding a very abundant *y* ion while suppressing others. Previously, we reported a similar phenomenon occurring at the *C*-terminus of citrulline residues and coined the term Cit effect. To confirm the presence of Cit effect in large proteomic datasets, we analyzed 293 peptides containing Cit residues based on the human proteome database mining work of Lee et al. (2018). The occurrence of Cit effect was found to be 44%. Comparing bond scissions at the amide linkage between Cit-Zzz (citrulline followed by a specified residue) to Aaa_1_-Aaa_2_ (Aaa can be any residue except Cit), 5 Cit-Zzz cleavages were significantly (CL = 95.0%) more frequent in > 85% of the cases in terms of relative sequential base beak occurrence. We used Pro effect to compare with Cit effect and obtained very similar results. On the other hand, our study showed that Cit effect is slightly inferior in the overall incidence to Pro effect (50% vs. 33%, CL = 95%) among deiminated peptides when Pro residues were also present in the sequence. Our results suggest that Cit effect is a characteristic feature and a possible biasing factor of deiminated peptides which can confirm the position of citrullination sites.

**Electronic supplementary material:**

The online version of this article (10.1007/s13361-019-02271-x) contains supplementary material, which is available to authorized users.

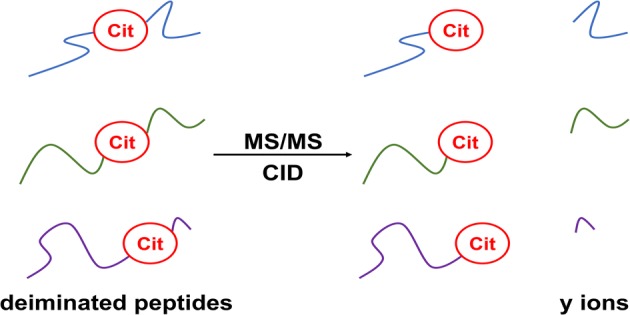

## Introduction

Protein citrullination or deimination is a post-translational modification (PTM) converting certain arginines to citrullines [1]. While citrullination was proved to be crucial in myelinization, immune and gene regulation, as well as in skin homeostasis, both hypercitrullination and hypocitrullination have been linked to pathological conditions in humans and animals too [2]. The strongest evidence is for the involvement in rheumatoid arthritis in which autoimmune response is triggered against citrullinated proteins [2–3]. Further associations have been found in multiple sclerosis [4–5], other neurodegenerative diseases [6–7], cancer [8–9], and heart failure [10]. The modification results in a loss of a positive charge and a small increase in molecular mass (0.9840 Da) per occurrence, which could lead to alteration in protein function and structure. Characterization of this low-abundance PTM by LC-MS experiments is still problematic [11–12]. A selective loss of isocyanic acid (HNCO) was previously observed for deiminated peptides upon collision-induced dissociation (CID) by Hao et al. [13] by which discrimination between citrullination and the isobaric transition, deamidation of Asn and Gln residues might have become possible via MS/MS. An alternative of CID, electron-transfer dissociation (ETD) technique usually results in better sequence coverage; however, a selective neutral loss for deiminated peptides in ETD is lacking [14]. Creese et al. used the neutral loss of isocyanic acid as a marker to select parent ions for a further ETD fragmentation process by supplemental activation (saETD) [15]. Although false positive hits were eliminated this way, false negative rate was still substantial; therefore, optimization of MS/MS identification of deiminated residues is still a key research field.

**Figure 1:**
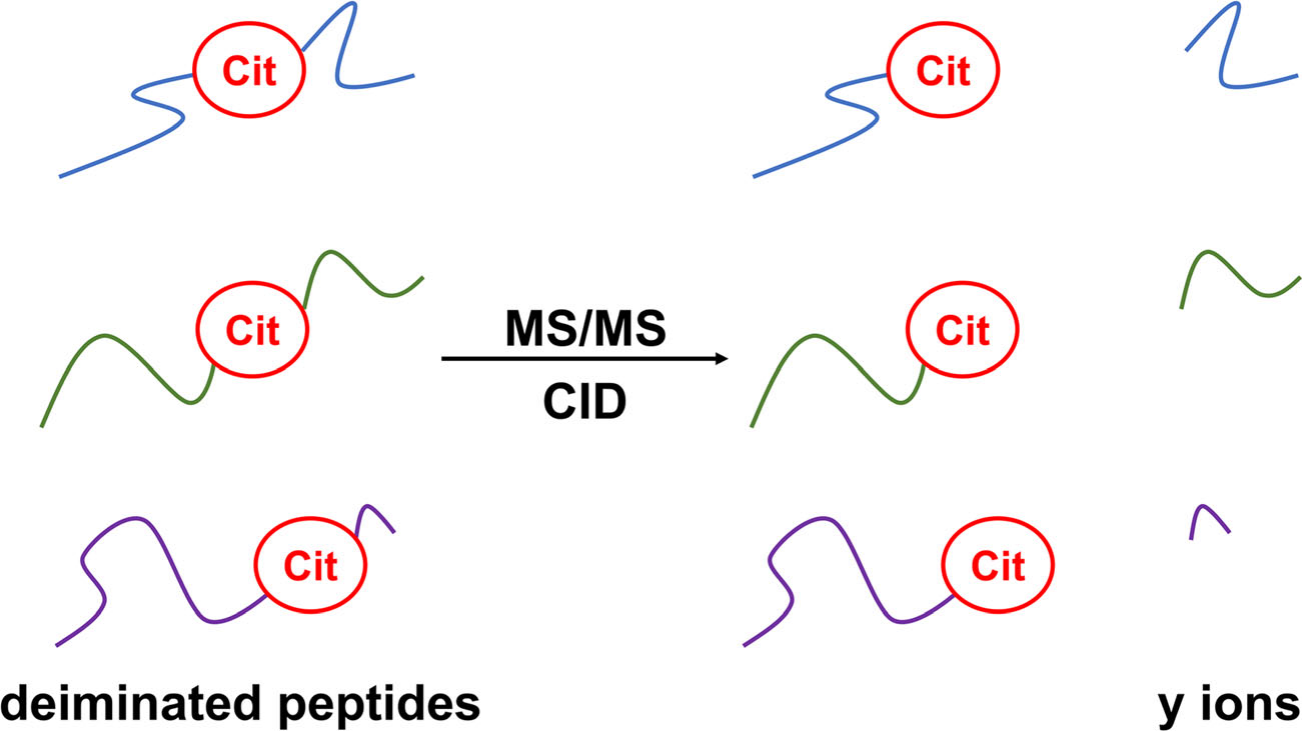
Schematic representation of Cit effect. Cit effect is characterized by an enhanced cleavage preference at the *C*-terminus of Cit residues upon collision-induced dissociation (CID) tandem mass spectrometry resulting in abundant y ions for tryptic peptides

Followed by enzymatic digestion of proteins, sample preparation, and chromatographic separation, peptides are usually identified via generation of *b* and *y* ion series as a result of amide cleavages [16–17] by CID. These ion series are characteristic to the sequence, and identification scores largely rely on them. Certain amino acids induce cleavages at their *N*- or *C*-termini with a higher preference, yielding the corresponding *b* or *y* ion with high intensity. These processes may hinder proper identification since they may suppress other valuable fragmentation routes. Proline effect which results in intensive peaks corresponding to the cleavage at the *N*-terminus of Pro residues (Aaa-Pro) due to the higher basicity of Pro has been long time known for tryptic peptides [18]. We previously reported a similar phenomenon for Cit residues upon fragmenting Cit-containing model peptides, but in these cases, the cleavage occurred at the *C*-terminus of Cit residues (Cit-Aaa) [19] (Figure 1) as in the case of the aspartic acid effect, where the preferred cleavage is at the Asp-Aaa linkage [18]. However, our previous sample size was not adequate (only 11 citrulline-containing synthetic model peptides were used), data were lacking on the overall occurrence, and the hypothesis was not tested on real proteomic samples before. Also, we could not determine whether the amino acid following Cit had an impact on Cit effect or not.

In 2018, a deep proteome mining was performed by Lee et al. exploring the human citrullinome for 30 tissues by a thorough examination of MS/MS spectral repositories with rigorous methods [20]. This group also exploited the presence of Cit-selective iminium ions in the MS/MS spectra of deiminated peptides to confirm the Cit-content of the hits. This was proved to be advantageous to filter out false positive (deamidated) peptides. Therefore, we used their published data of validated Cit-peptide sequences for our statistical evaluation omitting repetitive sequences to improve our knowledge concerning the nature of Cit effect.

## Experimental Procedures

### Experimental Design

The list of valid citrullinated peptides from proteomic data published by Lee et al. was used for our statistical evaluation (Table S5 in reference 20). Identical sequences originated from different human tissues were counted only once. Peptide sequences with matched and annotated MS/MS spectra were included only from PRIDE archive (data set identifier: PXD008970; filename: F095332_all_tissues_cit_extracted.dat) [21] via the use of PRIDE converter [22] and PRIDE inspector [23]. Peptides involved in our calculations were of tryptic origin with only few exceptions. No other selection criteria were used to avoid further possibility of sampling error. The 293 peptides included in our experiment are listed in Table S1.

For clarity and ease of interpretation, we defined the term sequential base peak (BP*). A sequential base peak is the most intensive sequentially informative ion (except for a few cases, these were *y* ions). Thus, sequences could be sorted either producing a BP* cleavage of a given amino acid linkage or not (dichotomous set). The proportion of the number of peptides where BP* peaks correspond to a given Zzz_1_-Zzz_2_ cleavage and the overall number of peptides containing that given amino acid connection were calculated (relative BP* occurrence or population proportion). Post-translationally modified amino acids were only taken into consideration if they were interconversions; e.g., deamidated Asn and Gln were considered Asp and Glu residues respectively. Please note that Aaa stands for any alpha amino acid while Zzz denotes a specific one.

### Theory

The statistical evaluation for determining the overall occurrence of Pro and Cit effect was done by a Wilson score method without continuity correction (CL = 95%) [24]. The approximate binomial calculation concerning Cit-Zzz and Aaa_1_-Aaa_2_ relative BP* occurrences for confidence intervals (CI) was also performed by Wilson score method without continuity correction (CL = 95%). This method is also applicable in case of low sample sizes’ proportions. The comparison between Cit-Zzz vs. Aaa_1_-Aaa_2_ proportions was carried out via an interval estimation for difference between two independent proportions using the Newcombe-Wilson method without continuity correction [25]. Aaa_1_-Aaa_2_ connections with an occurrence lower than *N* = 10 were excluded. This resulted in 172 Aaa_1_-Aaa_2_ links altogether. To eliminate the additive effect of the amino acid following citrulline on enhanced cleavage probability, we also compared the Cit-Zzz vs. Aaa-Zzz connections. This means, e.g., that Cit-Ala connections were investigated against all Aaa-Ala linkages to rule out that the effect is influenced solely by the presence of Ala residues. A positive value of percentage for both the lower and upper bound CI was accepted as statistically and practically significant and means the superiority of cleavage preference for the given Cit-Zzz vs. Aaa_1_-Aaa_2_. A negative percentage for the lower bound and a positive for the upper one were considered ambiguous (overlying CIs, or comparable cases). If both bounds had negative values, the Cit-Zzz cleavage was considered significantly less preferred than the comparator Aaa_1_-Aaa_2_. In order to compare our results with the earlier described, often observed Pro effect, we made our calculations for Zzz-Pro vs. Aaa_1_-Aaa_2_ as well.

## Results and Discussion

**Figure 2:**
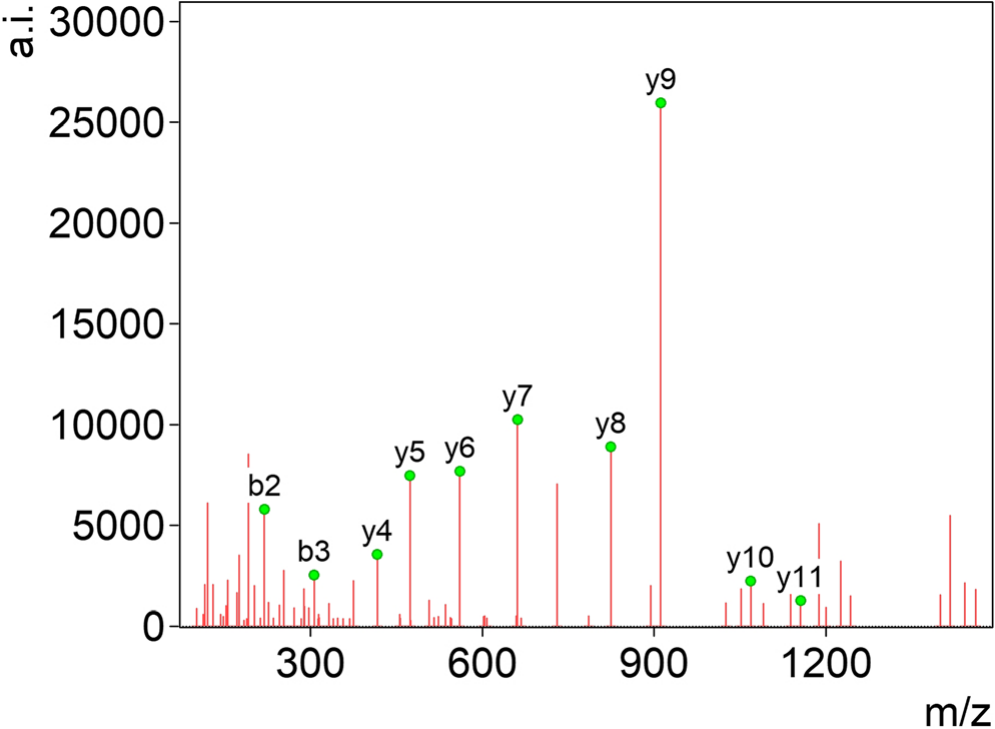
Tandem mass spectrum of the doubly protonated precursor of the peptide ^19^AFSSXSYTSGPGSR^32^ originated from keratin, type II, cytoskeletal 8 [20]. “X” is used for the one-letter abbreviation of Cit. The pronounced Cit effect is represented by a marked increase in the intensity of y_9_ fragment ion. In our experiments, all cases were considered to show Cit effect where the amide bond corresponding to a Cit-Zzz cleavage was a sequential base peak. Note that y_9_ fragment happens to be a real base peak here

In this work, sequential base peak (BP*) occurrences were determined for a number of natural deiminated peptides to obtain data on the amino acid effect of Cit. The relative BP* occurrence for all 293 peptides indicating Cit effect was found to be 44%. A typical spectrum showing a marked Cit effect is depicted in Figure 2. Data evaluation was carried out by the software “mMass” [26]. Six connections, Cit-Ala, Cit-Asp, Cit-Phe, Cit-Gly, Cit-Asn, and Cit-Ser amide bonds, were cleaved significantly more often to yield a BP* in > 85% of the cases compared with any cleavages between two given types of amino acids (Aaa_1_-Aaa_2_) included in our investigations (Table 1). For instance, Cit-Asp bond was found to be superior to 151/171 (87%) possible Aaa_1_-Aaa_2_ linkages. The most pronounced occurrence was observed for Cit-Gly and Cit-Ser. None of these six linkages was found to be inferior to that of any Aaa_1_-Aaa_2_. Five amide bonds including Cit-Ala, Cit-Asp, Cit-Gly, Cit-Asn, and Cit-Ser satisfied the criteria to be superior to their corresponding Aaa-Zzz category (e.g., Cit-Ala vs. Aaa-Ala, Cit-Asp vs. Aaa-Asp etc.) (Table S2), although Aaa does not often cover the whole set of amino acids possibly due to the low incidence of citrullinated peptides [12] and high sequence similarity of them. The amide bond at Cit-Glu was also found to be favorable for enhanced cleavage in 72% of possible linkages. It was found to be significantly superior than any other Aaa-Glu connection with the exception of Met-Glu. The increase of relative BP* vs. control varies greatly for the individual cases (Table S2).

**Table 1:** Summary of Cit-Zzz relative BP* occurrence vs. that of all linkages. Superior, inferior, and ambiguous indicate the proportion of cases in which the given Cit-Zzz was found superior, inferior or ambiguous vs. other possible amino acid connections (CL = 95.0%). Median strength of occurrence represents the median of the lower and upper Newcombe-Wilson confidence intervals for superior cases. Note that ambiguous means comparable cleavage preference or that a superior-inferior relationship cannot be determined

Cit effect						
Aaa1	Aaa2	sup%	inf%	amb%	Median strength of occurrence%	Median strength of occurrence%
Cit	Ala	87	0	13	21	66
	Asp	88	0	12	26	67
	Glu	71	2	27	7	48
	Phe	85	0	15	14	72
	Gly	94	0	6	36	74
	Leu	46	2	52	4	47
	Asn	87	0	13	18	75
	Gln	17	2	81	2	51
	Ser	94	0	6	34	77
	Thr	39	2	58	4	47
	Val	1	7	92	1	29

The median strength of occurrence for cases when Cit effect was superior to other cleavages is also shown in Table 1, represented by the median of the lower and upper value of Newcombe-Wilson confidence interval. These values demonstrate an average ability to produce BP* for the connections described above vs. any amide bonds. For example, the Cit-Gly amide bond is cleaved to yield the corresponding *y* ion as a BP* at least with an average of ~ (36–74)% more often than any other amide bonds. Interestingly, no BP* was observed for any Aaa-Cit connection further confirming that Cit-Zzz cleavage is preferred instead in case of tryptic peptides. Aspartic acid effect [19] was also not prevalent among deiminated peptides used in this study (Table S2).

Manual inspection also revealed that Cit effect was the most prominent fragmentation pathway when Cit was followed by a His or Pro residue due to an additive His and Pro effect resulting in suppressed fragment ion intensity for other ions, but these linkages seem to be underrepresented in Cit-containing peptides and were excluded from the statistical investigation.

**Figure 3:**
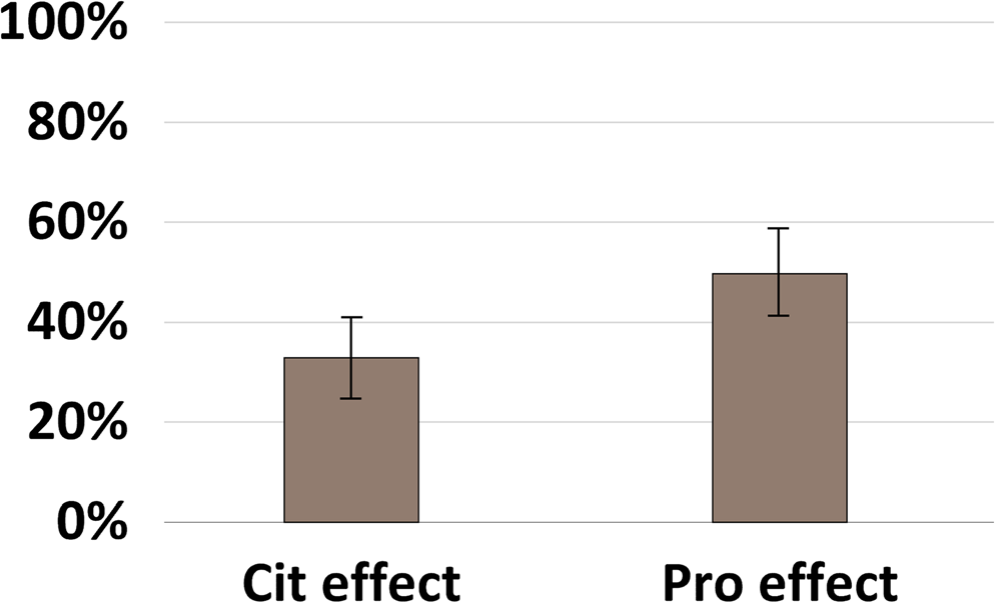
Comparison of the overall relative sequential base peak occurrences (BP*) for the deiminated sequences containing both Cit and Pro residues

By comparison of Cit and Pro effect, it was found that the latter was more frequent than the former regarding overall relative BP* occurrences, when both Cit and Pro residues were present in the sequences (50% vs. 33%; CI_Pro_ = 42–58%, CI_Cit_ = 26–41%) (Figure 3). The data on Aaa-Pro were also collected and summarized (Table 2). The results are very similar to that of obtained for Cit effect. Remarkably, cleavage of Gly-Pro amide bond was highly unfavorable to yield a BP* (population proportion was only found to be 1/28). Cleavage of Arg-Pro bonds was also suppressed (0/12) which may be explained by the superior basicity of Arg to Pro, resulting in the proton sequestration by the former to yield *b* ions instead of the corresponding *y* one.

**Table 2:** Summary of Aaa-Pro relative BP* occurrence vs. that of all linkages. Superior, inferior and ambiguous indicate the proportion of cases in which the given Aaa-Pro was found superior, inferior or ambiguous vs. other possible amino acid connections (CL = 95.0%). Median strength of occurrence represents the median of the lower and upper Newcombe-Wilson confidence intervals for superior cases. Note that ambiguous means comparable cleavage preference or that a superior-inferior relationship cannot be determined

Pro effect						
Aaa1	Aaa2	sup%	inf%	amb%	Median strength of occurrence%	Median strength of occurrence%
Ala	Pro	85	0	15	16	59
Gly		0	10	90	–	18
His		94	0	6	32	86
Leu		94	0	6	32	81
Pro		0	6	94	–	33
Arg		0	8	92	–	24
Ser		2	6	92	0	31
Thr		87	0	13	19	73
Val		88	0	12	24	83
Tyr		82	0	18	9	69

## Conclusions

A simplified cleavage preference matrix for deiminated peptides was created to confirm our hypothesis on Cit-Zzz connection as a site of favorable amide bond scission in deiminated peptides using CID fragmentation. We used the largest validated dataset [20] available for this purpose collected by Lee et al. (2018). To avoid statistical distortion, we applied Newcombe-Wilson method—which is less sensitive for low sample sizes—for evaluating the difference between the confidence intervals based on sequential base peak generation. Almost all analyzed peptides were of tryptic origin. Our statistically and practically significant results indicate that our previous hypothesis could be accepted for at least 5 connections that are Zzz = Ala, Asp, Gly, Asn, Ser. These cleavage preferences are superior in occurrence to most of the other ones and correlate with the natural occurrence of Cit-Aaa links [20] which may implicate that citrulline effect is a universal phenomenon or there is a significant bias toward the identification of the peptides carrying these types of connections. Another explanation could be the strict substrate preference of deimination, yielding a low number of citrullinated peptides in a physiological system despite the large number of tandem mass spectra used by Lee et al. On the other hand, Cit effect was found to be slightly inferior to Pro effect in terms of occurrence when both residues were present in the peptides. After careful optimization, Cit effect may be a promising candidate to improve the identification of citrullination sites by, e.g., collision energy-stepping or charge state manipulation along with the previously described loss of isocyanic acid and the use of Cit-selective iminium ion in proteomic analyses.

## Electronic supplementary material

ESM 1 (JPG 337 kb)

ESM 2 (JPG 302 kb)

ESM 3 (JPG 501 kb)

ESM 4 (XLSX 39 kb)

ESM 5 (XLSX 53 kb)

## Data Availability

Deiminated peptides that were used in our data analyses are listed in Table S1. Statistical calculations for all cases could be found in Table S2. By changing values *x* (number of favorable events, BP*) and *N* (number of events) in the cells highlighted in green, the Newcombe-Wilson lower and upper intervals are recalculated.
