# Supplementary figures and images for: Citrulline Effect Is a Characteristic Feature of Deiminated Peptides in Tandem Mass Spectrometry
Source: J Am Soc Mass Spectrom. 2019 Jul 12;30(9):1586–91. doi: 10.1007/s13361-019-02271-x (PMC6695478; doi:10.1007/s13361-019-02271-x)

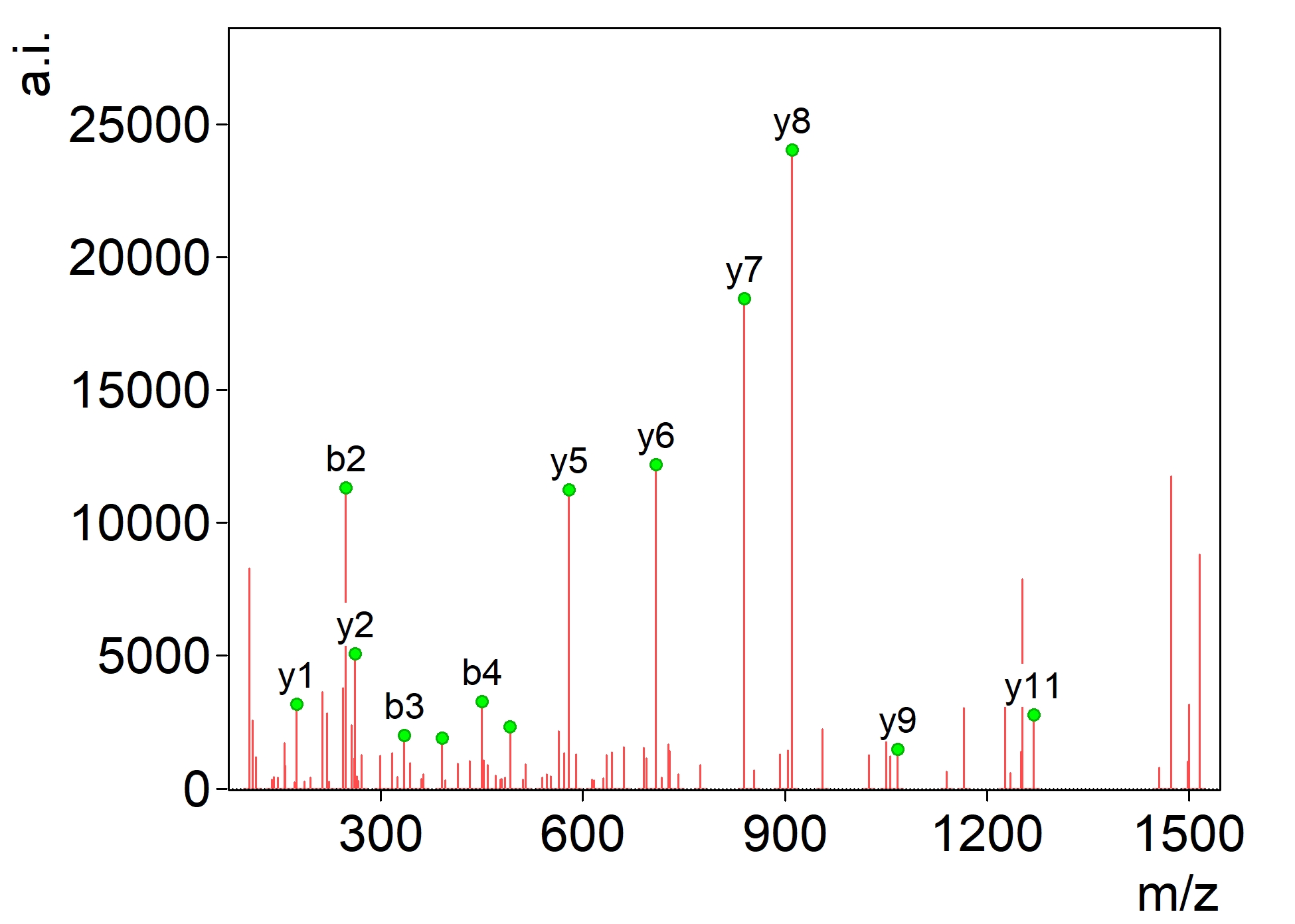

Supplement: Supplementary file 1 — (JPG 337 kb) [file 13361_2019_2271_MOESM1_ESM.jpg]

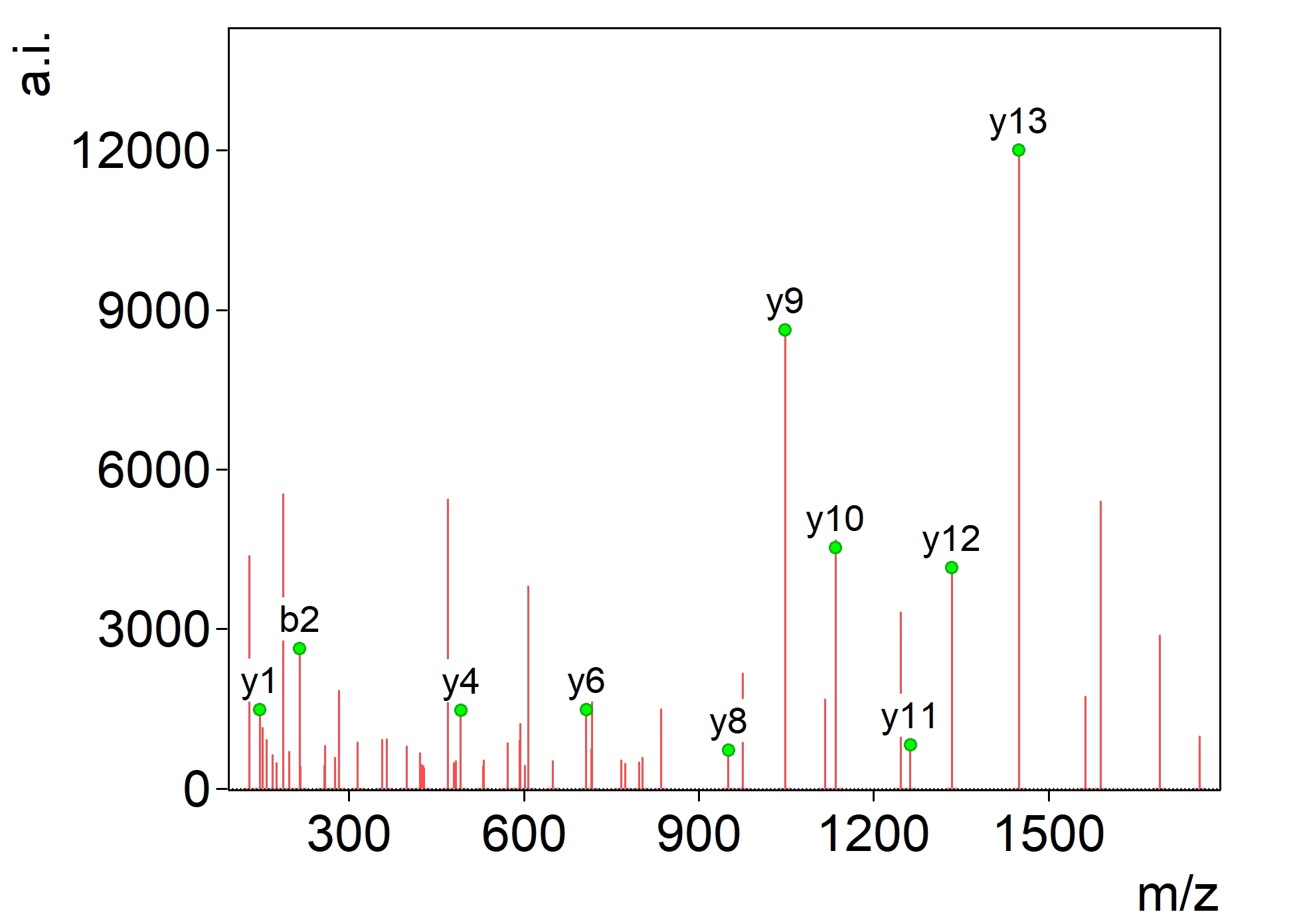

Supplement: Supplementary file 2 — (JPG 302 kb) [file 13361_2019_2271_MOESM2_ESM.jpg]

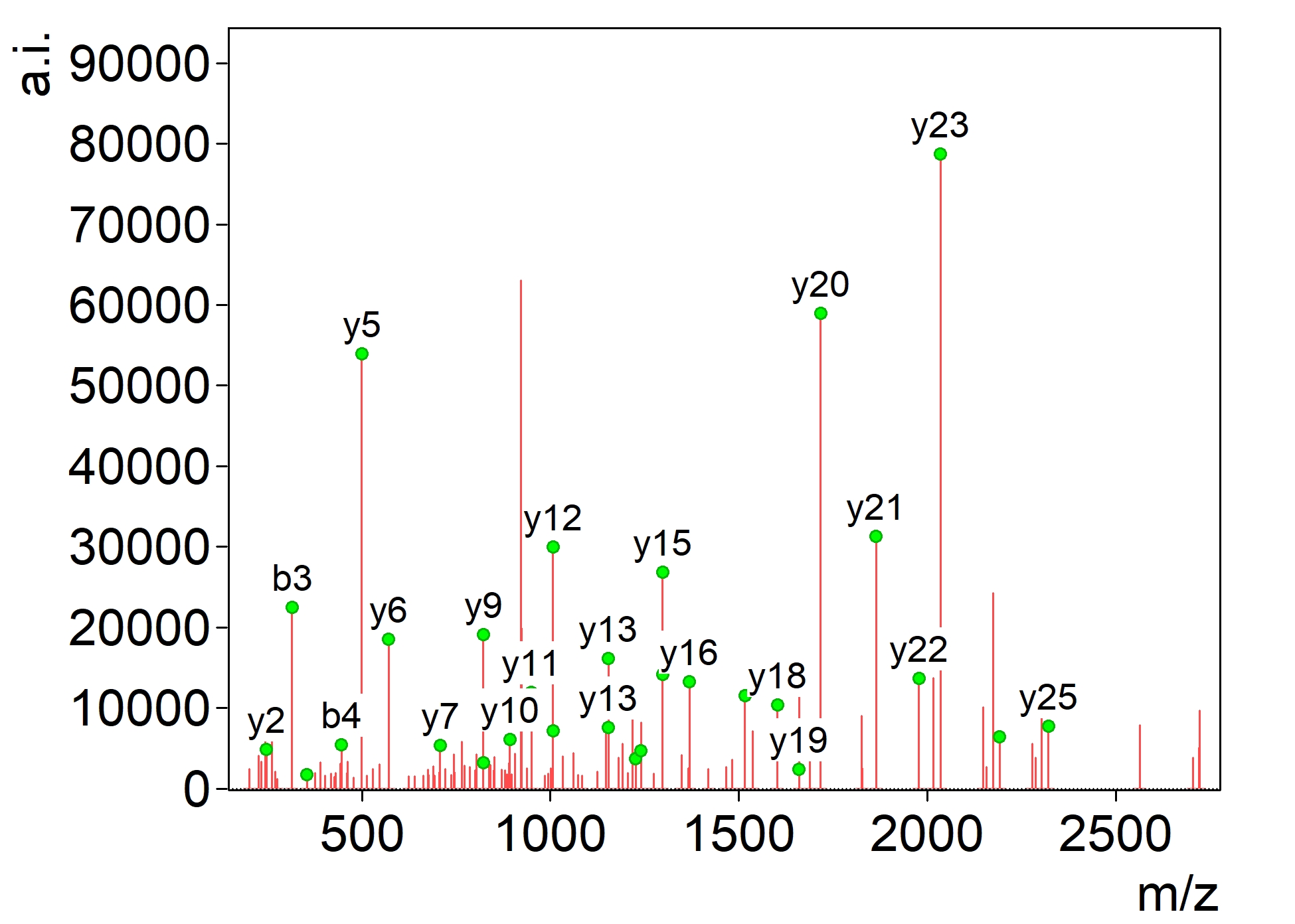

Supplement: Supplementary file 3 — (JPG 501 kb) [file 13361_2019_2271_MOESM3_ESM.jpg]
